# The Role of *MCM9* in the Etiology of Sertoli Cell-Only Syndrome and Premature Ovarian Insufficiency

**DOI:** 10.3390/jcm12030990

**Published:** 2023-01-28

**Authors:** Iulia Potorac, Marie Laterre, Olivier Malaise, Vlad Nechifor, Corinne Fasquelle, Orphal Colleye, Nancy Detrembleur, Hannah Verdin, Sofie Symoens, Elfride De Baere, Adrian F. Daly, Vincent Bours, Patrick Pétrossians, Axelle Pintiaux

**Affiliations:** 1 Department of Endocrinology, Centre Hospitalier Universitaire (CHU) de Liège, Domaine Universitaire Sart-Tilman, 4000 Liège, Belgium; 2 Department of Human Genetics, Centre Hospitalier Universitaire (CHU) de Liège, Domaine Universitaire Sart-Tilman, 4000 Liège, Belgium; 3 Department of Rheumatology, Centre Hospitalier Universitaire (CHU) de Liège, Domaine Universitaire Sart-Tilman, 4000 Liège, Belgium; 4 Department of Urology, Centre Hospitalier Universitaire (CHU) de Liège, Domaine Universitaire Sart-Tilman, 4000 Liège, Belgium; 5 Department of Pathology, Centre Hospitalier Universitaire (CHU) de Liège, Domaine Universitaire Sart-Tilman, 4000 Liège, Belgium; 6 Center for Medical Genetics Ghent, Ghent University and Ghent University Hospital, 9000 Ghent, Belgium; 7 Department of Gynecology, Centre Hospitalier Universitaire (CHU) de Liège, Domaine Universitaire Sart-Tilman, 4000 Liège, Belgium

**Keywords:** azoospermia, non-obstructive, premature ovarian failure, MCM9, puberty, genetics, Sertoli cell only syndrome

## Abstract

Infertility in couples is a common problem, with both female and male factors contributing to similar extents. Severe, congenital disorders affecting fertility are, however, rare. While folliculogenesis and spermatogenesis are generally orchestrated via different mechanisms, some genetic anomalies can impair both female and male gametogenesis. Minichromosome maintenance complex component 9 (MCM9) is involved in DNA repair and mutations of the *MCM9* gene have been previously reported in females with premature ovarian insufficiency (POI). *MCM9* is also an emerging cancer risk gene. We performed next-generation and Sanger sequencing of fertility and related genes and hormonal and imaging studies in a kindred whose members had POI and disordered spermatogenesis. We identified a homozygous pathogenic *MCM9* variant, c.394C>T (p.Arg132*) in three sisters affected by POI due to ovarian dysgenesis and their brother who had normal pubertal development but suffered from non-obstructive azoospermia. Testicular biopsy revealed Sertoli cell-only testicular histopathology. No evidence of early onset cancer was found in the homozygotic family members, but they were all young (<30 years) at the time of the study. In the male patient the homozygous *MCM9* variant led to normal pubertal development and hormonal levels but caused a Sertoli-cell-only syndrome with non-obstructive azoospermia. In the homozygous females studied, the clinical, hormonal, and gonadal phenotypes revealed ovarian dysgenesis consistent with previous reports. Active screening for potential colorectal and other cancer risks in the homozygotic *MCM9* subjects has been instigated.

## 1. Introduction

Infertility affects around 15% of couples [1], and the causes can be complex and multifactorial. Major strides have been made in recent years to identify the contribution of genetic factors to both male and female causes of infertility [2,3]. In females, premature ovarian insufficiency [POI], characterized by a decreased ovarian follicular reserve in those aged <40 years, affects >1% of women [4,5]. Primary amenorrhea due to hypergonadotropic hypogonadism lies at the severe end of the POI spectrum and its causes include monogenic diseases leading to anomalous follicular development or function [6]. Male causes are solely responsible for 20–30% of cases of infertility and, according to recent studies, global rates of male infertility range from 2.5 to 12% [7]. Non-obstructive azoospermia and related conditions that have a severe impact on fertility affect nearly 1% of the male population [8]. Whereas non-obstructive azoospermia was classically associated with chromosomal aneuploidy [Klinefelter syndrome] and microdeletions of Y chromosomal azoospermia factors, recent research has identified many novel genetic etiologies [9,10].

Some emerging monogenic disorders can affect both follicular function and spermatogenesis, such as biallelic mutations in the *minichromosome maintenance complex component 8 [MCM8*] gene [11,12]. MCM8 and MCM9 play important roles in DNA double-strand repair mechanisms [13] and disordered function of these proteins has been associated with primary gonadal failure and a potentially increased cancer risk [14,15,16]. Homozygous pathogenic variants in *MCM9* have previously been shown to lead to premature ovarian failure [17], but their role in spermatogenesis remains unclear.

Here, we report the characteristics of subjects with POI and non-obstructive azoospermia due to a homozygous pathogenic truncating *MCM9* variant. This is—to our knowledge—the first detailed clinical, hormonal, and gonadal description of a male individual with azoospermia due to a pathogenic variant of the *MCM9* gene.

## 2. Materials and Methods

Genomic DNA was extracted from blood leucocytes. It was enriched with the Sureselect All Exon v7 kit [Agilent Technologies] and was sequenced on a NovaSeq 6000 platform [Illumina]. Read alignment and variant calling were performed using a custom-developed bcbio-based pipeline. A minimum of 90% of all included genes have a coverage of at least 20×. Variants were interpreted and filtered using custom software. Data analysis of the 130 genes included in the ‘Disorders of sex development—Primary ovarian insufficiency—Hypogonadotropic hypogonadism’ gene panel version 5 [Center for Medical Genetics, University of Ghent, Belgium] was performed in the first two sisters [IV-1 and IV-5]. The pathogenic *MCM9* variant was later sequenced using Sanger sequencing in the other family members [IV-2, IV-3, and IV-6]. For IV-3, genomic DNA was enriched by the HyperCap EZ Prime Choice Probes [Roche] and the 43 genes included in the ‘Osteogenesis imperfecta/Osteoporosis’ panel [Center for Medical Genetics, University of Ghent, Belgium] were sequenced using Illumina’s sequencing by synthesis [SBS] technology. Variants reported have been confirmed by Sanger sequencing. The *COL1A2* variant detected in IV-3 was sequenced using Sanger sequencing in the other family members [III-1, III-2, IV-1, IV-2, IV-5, IV-6]. Primers used for *MCM9* and *COL1A2* Sanger sequencing and technical details of next-generation sequencing panels are available on request. All subjects and/or parents provided informed written consent for genetic studies and other examinations.

**Figure 1:**
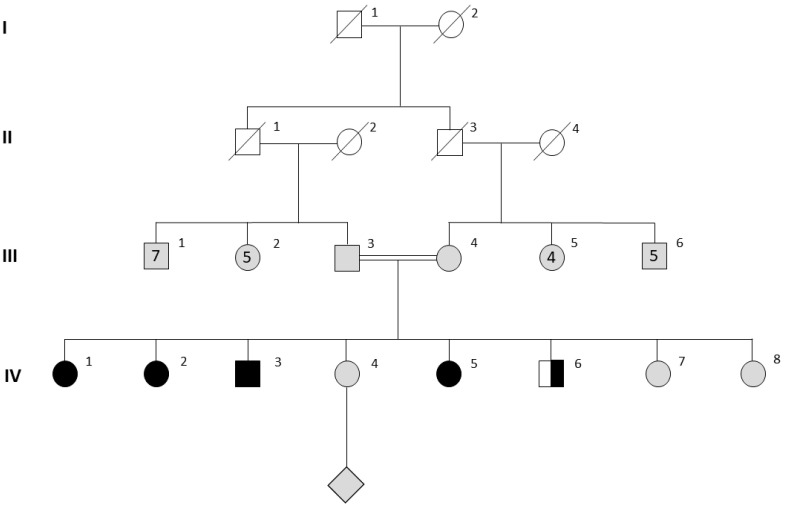
Pedigree of a consanguineous kindred with *MCM9* pathogenic variant. Males and females are designated by squares and circles, respectively. In generation III, the numbers of offspring are indicated by Roman numerals inside the squares/circles, the diamond refers to multiple offspring of individual IV-4 Homozygotes for the *MCM9* pathogenic variant are shown in black, heterozygotes in black and white, untested subjects in grey and deceased individuals in white with a strikethrough.

Clinical data on the included subjects were as follows: two sisters aged 24 [IV-1] and 17 years [IV-5] were referred for investigation of primary amenorrhea. The sisters belong to a consanguineous family of Middle Eastern origin, their parents being paternal first-degree cousins. The parents had eight children [six daughters and two sons—Figure 1]. Patient IV-1 reported that in her late teens her breast development was absent, but she had normal axillary and pubic hair. She received hormonal treatment [estradiol valerate 2 mg and norgestrel 0.5 mg] between the ages of 18 and 20 at another center. This led to some breast development and the onset of menses but after stopping treatment, her amenorrhea returned. On clinical examination when she presented to our center, she had minor breast development, and normal axillary and pubic hair. The younger sister IV-5, who was naïve to hormonal treatment, had absent breast development, normal axillary and pubic hair, and no other notable physical features on physical examination. A third sister, IV-2 presented elsewhere with the same clinical phenotype [absent breast development and primary amenorrhea] as IV-1 and IV-5. One other sister, IV-4, had a normal puberty and had conceived naturally. The other two remaining sisters were still pre-pubertal at the time of presentation. Two brothers were 19 [IV-3] and 15 [IV-6] at the time of presentation, and both had entered puberty normally and were clinically well virilised.

Complete hormonal analyses were performed in the six post-pubertal subjects. Whenever appropriate, imaging studies such as pelvic ultrasound and MRI, scrotal ultrasound, and bone densitometry were performed. Genetic analyses were requested. As osteoporotic values were found in the male index patient, an osteoporosis gene panel was requested. An open testicular biopsy was performed on patient IV-3 and two testicular fragments were sampled. Staining with hematoxylin-eosin, trichrome, orcein, placental alkaline phosphatase [PLAP], sal-like protein 4 [SALL4], inhibin, androgen receptor, actin, and calretinin immunostaining were performed.

## 3. Results

### 3.1. Clinical Characteristics in Females

Hormonal work-up in female subjects IV-1 and IV-5 revealed hypergonadotropic hypogonadism [Table 1] and very low AMH levels. A pelvic MRI found a hypotrophic uterus in subject IV-1, who had previously been treated with hormone replacement therapy. No uterus and a hypoplastic vagina were identified in the younger, treatment naïve sister IV-5. The ovaries could not be visualised in either sister. Severe osteoporosis was present in both patients, with Z-scores of −4.2 in the lumbar spine and 3.7 in the femoral neck for IV-1 and −4.8 in the lumbar spine, and −2.9 in the femoral neck in IV-V. Hormonal substitution with transdermal oestradiol 1.5 mg daily, then 2.25 mg daily was started in both sisters and after six months, dydrogesterone 10 mg daily, 10 days/month was added. At the last follow-up 18 months later, bone mineral density had improved in both sisters with Z-scores of −3.2 [from −4.2] in the lumbar spine and −2.9 [from −3.7] in the femoral neck in the elder sister IV-1 and −3.0 [from −4.8] in the lumbar spine and −1.7 [from −2.9] in the femoral neck in subject IV-5. Pelvic MRI in subject IV-5 showed her uterus had grown and now measured 5 cm in length and the vagina was normal in appearance.

### 3.2. Genetic Investigations

Genetic investigations revealed a normal karyotype and the absence of the fragile X syndrome in subjects IV-1 and IV-5. Ovarian autoimmunity was absent. At that point, a genetic panel analyzing genes involved in POI was completed. The results revealed a previously described [17] homozygous pathogenic variant of the *MCM9* gene, c.394C>T [p.Arg132*] in both sisters. This rare variant has a total allele frequency of 0.0006573% in gnomAD v3.1.2 and leads to a premature stop codon predicted to result in nonsense-mediated decay (NMD). Upon establishing the family tree, a third sister, IV-2, aged 23 was also diagnosed with primary amenorrhea. Her clinical presentation, hormonal work-up, and bone densitometry results were the same as in her two sisters. She was also homozygous for the familial *a* gene variant, c.394C>T [p.Arg132*; Table 1].

### 3.3. Male Characteristics and Testicular Histopathology

**Figure 2:**
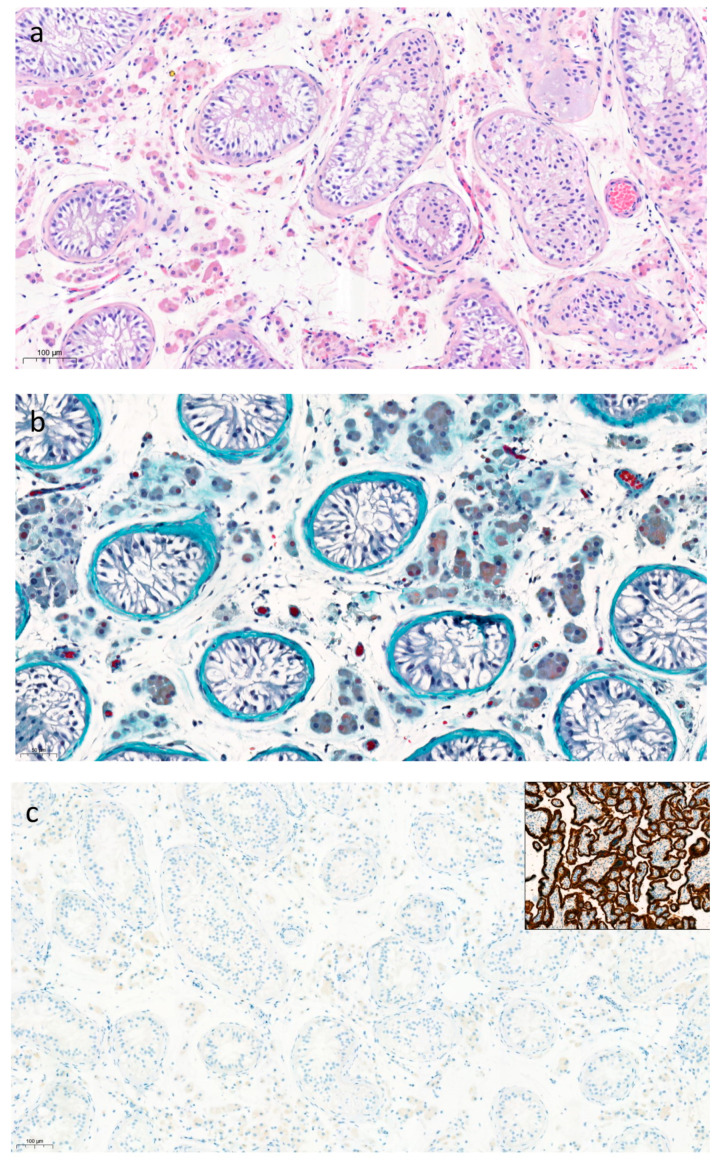

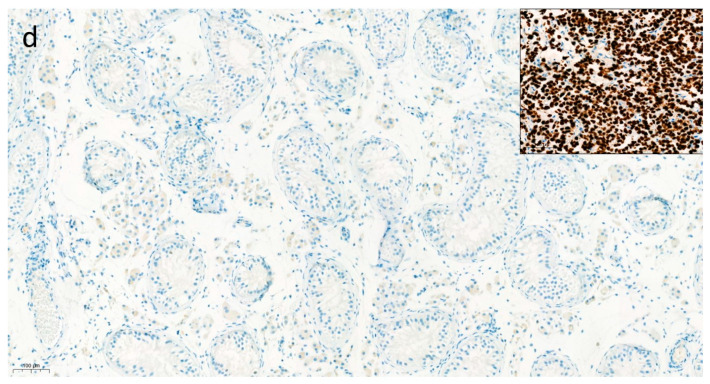
Histopathological analysis of testicular biopsy in subject IV-3. Seminiferous tubules with slight thickening and hyalinization of the basement membrane [(**a**). hematoxylin and eosin staining, ×10, (**b**). trichrome staining, ×20]; Absence of germ cells on immunohistochemistry for PLAP (with positive placenta control sample in inset) (**c**) and SALL4 (with positive yolk sac tumor control in inset) (**d**).

The 19-year-old male subject IV-3 presented because of fertility concerns following a spermogram that had revealed azoospermia. On examination, he had normal pubertal development and a repeat sperm analysis confirmed azoospermia. His hormonal work-up was normal with an inhibin B level in the low-normal range [117.4 ng/L, normal range: 105–439]. Scrotal ultrasound revealed the presence of testes of small size [26–30 mm maximum length] and of normal echogenicity. There were no anomalies suggestive of obstructive azoospermia. On initial genetic analysis, his karyotype was 46, XY; Y chromosome microdeletions were absent. He was found to be homozygous for the familial *MCM9* pathogenic variant. A testicular biopsy revealed Sertoli cell-only syndrome with a complete absence of germ cells (Figure 2). The basement membranes surrounding the seminiferous tubules were slightly thickened. Staining for PLAP and SALL4 was completely absent (Figure 2). Leydig cells and seminiferous tubules stained positively for androgen receptors and inhibin, while Leydig cells were positive for calretinin. His younger brother, subject IV-6, underwent clinical studies. His hormonal work-up was normal, and he was a heterozygous carrier of the familial *MCM9* variant.

### 3.4. Bone Phenotype and Investigations

Due to the severe osteoporosis in his sisters, patient IV-3 underwent bone densitometry. Bone mass was also low with Z-scores of −3.1 in the lumbar spine and −1.7 in the femoral neck. As the patient had normal androgen levels, a genetic panel for osteoporosis was requested. The results did not reveal any pathogenic or likely pathogenic [class 4–5] variants in the genes studied. A heterozygous variant of uncertain significance in the *COL1A2* gene [c.106G>A, p.Gly36Arg] was found; *COL1A2* codes for the alpha2 chain of type I collagen. In light of the early onset familial osteoporosis and the results of the genetic analyses, bone densitometries were also performed in the parents and in the younger brother, subject IV-6. The father, aged 48, also had osteoporosis [lumbar spine T-score −3.0, femoral neck T-score −2.3]. He had lost 5.5 cm in height [based on his reported adult height]. The mother, aged 42, had minor lumbar spine osteopenia [Z-score −1.2] with a normal femoral neck bone mass. The *COL1A2* variant did not fully segregate with the bone pathology: the father was wild-type and the mother was a heterozygous carrier. Subject IV-6 had lumbar spine and femoral neck Z-scores of −1.1 and −2.2, respectively; he was heterozygous for the *COL1A2* variant.

## 4. Discussion

The number of known genetic causes of POI has grown progressively in recent years [18]. While the most common genetic anomalies responsible for POI remain Turner syndrome and fragile X syndrome, several other genes with roles in ovarian development and function are now known to be involved. Ovarian dysgenesis, which lies at the severe end of the POI spectrum, is often monogenic in origin [19]. The genes responsible for this phenotype play important roles in primordial follicle formation, activation, development, and maturation or in premature apoptosis of the follicular reserve [19]. We report a family with a homozygous pathogenic *MCM9* variant consisting of three sisters with ovarian dysgenesis and their brother, who is the first male patient with this pathogenic variant and non-obstructive azoospermia due to Sertoli cell-only syndrome that was proven on testicular biopsy.

Non-obstructive azoospermia can be due to Sertoli cell-only syndrome, maturation arrest, or severely limited spermatogenesis [20]. In Sertoli cell-only syndrome, germ cells are absent and Sertoli cells are found lining the seminiferous tubules. Testicular volume is lower than in patients with obstructive azoospermia or maturation arrest, the basement membranes are thicker and the tubular lumina are narrower [21]. All of these features were found in the male patient with the homozygous *MCM9* variant. As he had normal FSH values and a low normal inhibin B, some limited focal spermatogenesis cannot be excluded. 

So far, less than a dozen cases of POI in relation to *MCM9* pathogenic variants have been reported [16,17,22,23]. The first cases were found to have short stature [17], but this was not confirmed in other cases, and short stature was not seen in the current family. In fact, the height of one of our female patients surpassed her target height as calculated based on the mid-parental heights. The *MCM9* variant found in the current family [c.394C>T, p.Arg132*] is the same as the one reported by Wood-Trageser et al. [17]. Subsequently, another POI case with this pathogenic variant was reported by Jolly et al. [24]. These various families have a similar geographic origin, so they may be members of an extended family or there may be a founder effect in their local sub-population. The genetic modification leads to a truncated protein, which is most likely dysfunctional. 

MCM9 forms a complex with MCM8 and plays an important role in DNA mismatch repair mechanisms [25]. Genetic anomalies in these genes negatively impact homologous recombination-mediated DNA repair both during meiosis and during mitosis [15]. As the former occurs during the formation of gametes, it is likely that gametogenesis is impaired by *MCM9* variants, thus explaining the ovarian dysgenesis and the Sertoli cell-only syndrome. Two whole-exome sequencing studies analysing the genetic causes of azoospermia or severe oligospermia reported homozygous variants of the *MCM9* gene [9,10]. In one of these studies, the same *MCM9* variant as in the currently reported family was found. Additionally, a case of severe oligo-terato-astheno-zoospermia has been reported in a patient carrying a heterozygous pathogenic *MCM9* variant [16]. However, the histopathological basis of the non-obstructive azoospermia has never been reported previously in *MCM9* mutated subjects. 

Our results indicate that azoospermia is probably due to the nearly complete absence of germ cells, which corresponds to results in animal models. Knock-out or gene-trap murine models for *MCM8* and *MCM9* have been studied [15,26]. These mice models exhibit defects in homologous recombination-mediated DNA double-strand break repair and mismatch repair. In terms of fertility, both male and female *MCM8* KO mice are sterile [15]. In male mice, spermatogenesis is blocked in meiotic prophase I, while female mice have arrested primary follicles. In gene trap mice lacking *MCM9*, females are sterile with ovaries devoid of oocytes or markedly depleted in terms of primordial follicles [15,26]. Males maintain some reproductive capacity with much lower production of spermatozoa [15] and have progressive, severe germ cell loss [25]. Testicular histology in mice reveals some seminiferous tubules with relatively normal spermatogenesis and some with a Sertoli cell-only aspect [27]. The mechanism underlying the reproductive phenotype appears to be the reduced primordial germ cell proliferation, as a way to protect genome integrity in germ cells [27].

Murine *MCM9*-deficient models exhibit genome instability and are predisposed to the development of different types of cancers [hepatocellular carcinoma, ovarian tumors] [26]. Moreover, *MCM8* and *MCM9* KO mice are predisposed to hematopoietic proliferation anomalies with the development with the age of myeloid tumors that are similar to myelodysplastic syndromes in humans [14]. In humans, pathogenic *MCM9* variants appear to be associated with hereditary mixed polyposis and early onset colorectal cancer, causing a Lynch-like syndrome [16,28]. A case of clear cell cervical cancer and a germ cell tumor has also been reported [16]. Increased colorectal polyp and cancer risk at a young age appears to be associated mainly with bi-allelic [homozygous] pathogenic *MCM9* variants, while mono-allelic individuals carrying *MCM9* variants have a wider age range or variable penetrance, as noted by Goldberg et al. [16]. In the current family, there was no established history of cancer in the heterozygotic parents. As the homozygotic *MCM9* variant subjects are all <30 years of age, they may have been too young to have developed cancers at the time of the study. In the absence of any validated screening guideline for patients with *MCM8/MCM9* pathogenic variants, we propose a program of regular colorectal and cervical screening and vigilance for the onset of other tumors for these patients.

Osteoporosis in female POI patients is most likely secondary to severe and prolonged hypogonadism, as a direct role for *MCM9* pathogenic variants in the bone has not been described. Furthermore, after 18 months of hormonal treatment and calcium and vitamin D supplementation, the bone mass in two patients improved significantly, both at the lumbar spine and at the femoral neck. However, the osteoporosis in the brother with Sertoli cell-only syndrome remains unexplained, as his hormonal profiles at presentation were normal. A genetic panel for over 40 genes involved in osteoporosis was analysed in the family, revealing a VUS in *COL1A2*. As variants in this gene have previously been reported in relation to early onset osteoporosis without an osteogenesis imperfecta phenotype [29,30], we considered this as a potential candidate to explain the bone phenotype. However, this *COL1A2* VUS was inherited from the mother who had relatively unremarkable bone mineral density and the father with osteoporosis was wild-type, indicating a lack of segregation of the *COL1A2* variant with the bone phenotype. There is little information in the literature linking non-obstructive azoospermia to bone disease. However, one group found that the expression of vitamin D 25-hydroxylase was lower in the testicular samples from azoospermic men compared to normal controls [31]. These patients were also found to exhibit osteopenia and osteoporosis more frequently than controls despite a seemingly normal testosterone production [31]. However, as osteoporosis was also found in the younger brother and the father, who were heterozygous for the *MCM9* pathogenic variant, this argues against a mechanism via homozygous *MCM9* pathogenic variants leading to loss of bone mass in the family. Further assessments of the bone phenotype in the family during long-term follow-up may reveal other risk factors as new genetic causes of osteoporosis are identified.

## 5. Conclusions

Homozygous pathogenic *MCM9* variant in the human male is associated with normal pubertal development and hormonal levels but leads to a Sertoli cell-only syndrome causing non-obstructive azoospermia. Larger studies are required to confirm this link and to define the penetrance of the phenotype in these patients. In three homozygous female the clinical, hormonal, and gonadal phenotypes were severe and consistent with previous reports of POI, albeit that short stature was not seen. At this time no colorectal or other cancerous lesions have become apparent, but specific screening will continue.

## Figures and Tables

**Table 1:** Clinical, hormonal, genetic, and bone characteristics in subjects with *MCM9* pathogenic variant.

	IV.1	IV.5	IV.2	IV.3	IV.6	III.3	III.4
**Gender**	female	female	female	male	male	male	female
***MCM9* status**	c.394C>T, p.Arg132* homozyg.	c.394C>T, p.Arg132* homozyg.	c.394C>T, p.Arg132* homozyg.	c.394C>T, p.Arg132* homozyg.	c.394C>T, p.Arg132* heterozyg.	NA	NA
**Pubertal status**	Absent	Absent	Absent	Normal	Normal	Normal; fertile	Normal; fertile
**FSH (U/L)**	117	112	106	5	4.5	NA	NA
**LH (U/L)**	41	24	56	4.8	3.8	NA	NA
**Estradiol (ng/L)**	<24	<24	<24	31	NA	NA	NA
**Testosterone (nmol/L)**	1.36	1.2	0.5	23.7	28.8	NA	NA
**L spine Z-score**	B/L 18 mo. −4.2 −3.2	B/L 18 mo −4.8 −3.0	−3.4	−3.1	−1.1	−3.0	−1.2
**Fem neck Z-score**	B/L 18 mo. −3.7 −2.9	B/L 18 mo −2.9 −1.7	−2.6	−1.7	−2.2	−2.3	−0.7
**Sperm analysis**	-	-	-	Non-obstructive azoospermia	ND	ND	-
***COL1A2* status**	c.106G/G	c.106G/G	c.106G/A	c.106G/A	c.106G/A	c.106G/G	c.106G/A

B/L = Baseline; Fem neck = femoral neck; heterozyg = heterozygous; homozyg = homozygous; L spine = lumbar spine; mo = month; NA = not available; ND = not done.

## Data Availability

Data reported in this study can be made available upon reasonable request to the authors.
